# On the Influence of the Epiglottis in the Formation of the Voice

**Published:** 1844-09

**Authors:** 


*On the Influence of the Epiglottis in the Formation of the Voice.*—P. Mayer refutes, in an article on the human voice, the assertion of Magendie relative to the inability of the epiglottis during deglutition. He here proves that this organ serves not only for deglutition, but even for the vocalization of sounds. It is easy to be convinced on one's own person, that in low sound the epiglottis rises and rests on the back of the tongue; but the most remarkable fact is, that the epiglottis should become altogether flat; in the high sounds, on the contrary, it becomes horizontal, and, what is the essential point, its two sides roll inwards, so that the epiglottis forms a kind of horn, which receives the sound and condenses the sonorous undulations. Moreover, the epiglottis vibrates at the instigation of the *chordæ vocales*, and these vibrations depend on its greater tension. It is probable that there is an exact relation between the tension of the chords and that of the epiglottis; these motions still serve to strengthen the sound.—*Forceps.*

